# GPR176 Promotes Cancer Progression by Interacting with G Protein GNAS to Restrain Cell Mitophagy in Colorectal Cancer

**DOI:** 10.1002/advs.202205627

**Published:** 2023-03-11

**Authors:** Junwei Tang, Wen Peng, Jiangzhou Ji, Chaofan Peng, Tuo Wang, Peng Yang, Ji'ou Gu, Yifei Feng, Kangpeng Jin, Xiaowei Wang, Yueming Sun

**Affiliations:** ^1^ Department of General Surgery The First Affiliated Hospital of Nanjing Medical University Nanjing Jiangsu 210029 P. R. China; ^2^ Colorectal Institute of Nanjing Medical University Nanjing P. R. China; ^3^ The First School of Clinical Medicine Nanjing Medical University Nanjing P. R. China

**Keywords:** autophagy, GPCR, growth, homology modeling, mitochondria

## Abstract

GPR176 belongs to the G protein‐coupled receptor superfamily, which responds to external stimuli and regulates cancer progression, but its role in colorectal cancer (CRC) remains unclear. In the present study, expression analyses of GPR176 are performed in patients with colorectal cancer. Genetic mouse models of CRC coupled with Gpr176‐deficiency are investigated, and in vivo and in vitro treatments are conducted. A positive correlation between GPR176 upregulation and the proliferation and poor overall survival of CRC is demonstrated. GPR176 is confirmed to activate the cAMP/PKA signaling pathway and modulate mitophagy, promoting CRC oncogenesis and development. Mechanistically, the G protein GNAS is recruited intracellularly to transduce and amplify extracellular signals from GPR176. A homolog model tool confirmed that GPR176 recruits GNAS intracellularly via its transmembrane helix 3‐intracellular loop 2 domain. The GPR176/GNAS complex inhibits mitophagy via the cAMP/PKA/BNIP3L axis, thereby promoting the tumorigenesis and progression of CRC.

## Graphical abstract

GPR176 recruits GNAS and activates the cAMP/PKA/BNIP3L axis, thereby inhibiting mitophagy and promoting CRC progression.

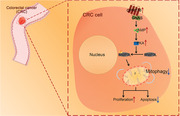

## 1 Introduction

Colorectal cancer (CRC) is a common gastrointestinal malignancy with 1.8 million new cases and 881000 deaths estimated in 2018, placing it among the top five most prevalent cancers worldwide.^[^ ^1^ ^]^ In developed countries, the incidence and mortality of CRC rank the third and second highest among all cancers, respectively.^[^ ^2^ ^]^ Currently, with improvements in comprehensive treatments, such as neoadjuvant therapy, radical surgery, postoperative radio‐chemotherapy, and immunotherapy, the prognosis of CRC patients has improved. However, for those in stages III and IV, according to the American Joint Committee on Cancer staging system, the five‐year survival rates are 71% and 14%, respectively, which are relatively low.^[^ ^3^ ^]^ Therefore, it is necessary to investigate the underlying mechanisms of CRC occurrence and development, explore biomarkers for early CRC diagnosis, and develop effective treatments.

Recently, G protein‐coupled receptors (GPCRs) have become the focus of molecular‐targeted therapy.^[^ ^4^ ^]^ As seven‐transmembrane receptors, GPCRs are a superfamily of cell surface receptors involved in human disease.^[^ ^5^ ^]^ In response to extracellular stimuli, GPCRs are activated, changing their structural conformation to expose their binding sites to the downstream G protein subunit G*α* and transduce signals intracellularly.^[^ ^6^ ^]^ However, only 50 to 60 GPCRs have been implemented as drug targets, the majority of which remain in an orphan state, and research on GPCRs in CRC remains scarce. Nonetheless, using GPCRs as an entry point may increase options for CRC treatment.

Mitophagy, an evolutionarily conserved cellular process that eliminates senescent or damaged mitochondria to maintain cellular energy, usually serves as a tumor‐suppressive system.^[^ ^7^ ^]^ However, the impact of mitophagy on cancer progression remains controversial given that mitophagy also plays a tumor‐promoting role depending on cellular context.^[^ ^8^ ^]^ For example, mitophagy mediated by NIX, a new oncogenic KRAS effector, promotes cell proliferation and metastasis in pancreatic ductal adenocarcinoma.^[^ ^9^ ^]^ Conversely, in hepatic cancer, FUNDC1‐mediated mitophagy suppresses hepatocellular carcinoma (HCC) initiation.^[^ ^10^ ^]^ Furthermore, few studies have focused on the role of mitophagy in the oncogenesis and progression of CRC. Although Ziegler et al. reported that mitophagy induced antitumor immunity and controlled the CD8^+^ T cell‐based adaptive immune response in CRC,^[^ ^11^ ^]^ more studies are needed to elucidate the relationship between mitophagy and CRC development.

Canonically, second messenger cAMP, produced following GPCR activation, plays a fundamental role in cellular metabolism as an allosteric activator of PKA.^[^ ^12^ ^]^ The cAMP/PKA pathway modulates mitophagy via the phosphorylation of PKA substrates.^[^ ^13^ ^]^ As a member of GPCRs family, GPR176 is a cell surface receptor involved in responses to hormones, growth factors, and neurotransmitters.^[^ ^14^ ^]^ GPR176 has agonist‐independent constitutive activity, which allows it to reduce the synthesis of cAMP without ligand involvement.^[^ ^15^ ^]^ Until now, the role of GPR176 in CRC has not been reported.

In this study, we investigated the functions and mechanisms of GPR176, particularly regarding the role in mitophagy in CRC. We found that GPR176 was upregulated in CRC tumors and responsible for oncogenesis and development both in vitro and in vivo. Moreover, subsequent validation experiments confirmed a correlation between GPR176 and mitophagy. Mechanistically, abnormal GPR176 expression enhanced the cAMP/PKA pathway, which then phosphorylated BNIP3L and abrogated its ability to induce mitophagy. Meanwhile, a co‐immunoprecipitation (Co‐IP) assay indicated that the G protein subunit encoded by *GNAS* binds to GPR176 to exert a signal‐trading role in cells, eventually boosting the oncogenesis and development of CRC. These findings suggested that GPR176 is a potential therapeutic target for CRC.

## 2 Results

### 2.1 GPR176 was Upregulated in CRC and Positively Correlated with Poor Prognosis

**Figure 1:**
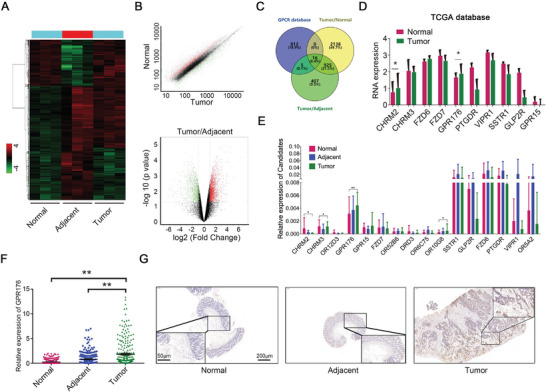
Increased GPR176 was identified in colorectal cancer. A) The cluster analysis of dysregulated mRNA in tissues obtained from CRC patients. B) Significant different expression mRNA in tumor tissues, corresponding adjacent tumor tissues, and normal tissues. C) Overlapped results combining high throughput screening data with GPCR database. D) TCGA analysis of candidate GPCRs expression in colorectal cancer tissues versus normal tissues, including 51 normal and 379 tumor samples. E) Relative expression of candidate GPCRs between normal, adjacent normal, and cancer tissues analyzed by quantitative reverse transcription‐PCR (qRT‐PCR), *n* = 20. F) Large cohort validated the expression of GPR176 in tissue samples obtained from CRC patients, *n* = 249. G) Immunohistochemistry of GPR176 in histologic section of CRC patients. The representative images were shown with scale bars of 200 µm and magnified scale bars of 50 µm. Data was presented with mean±SD, **p < 0.01 and *p < 0.05.

In order to screen all GPCRs in CRC, a high‐throughput transcription profile containing tumor tissues, paired normal tissues, and adjacent tissues was constructed to investigate differentially expressed genes (DEGs) (**Figure** **1**A). Dysregulated genes were identified (|log_2_FC| >1, *p* < 0.05) (Figure 1B). Overlapping these DEGs with human‐verified GPCRs obtained from the GPCR database (https://gpcrdb.org/), 16 GPCRs were filtered out, with 6 upregulated and 10 downregulated (Figure 1C). We then assessed the expression patterns of these 16 genes in the Cancer Genome Atlas (TCGA) database. Except 6 GPCRs without express data in TCGA, 10 GPCRs were analyzed. Among them, only *CHRM2* and *GPR176* were overexpressed in tumors (Figure 1D). A qRT‐PCR was used to quantify these 16 GPCRs in 20 pairs of CRC samples. The results confirmed the increased level of *GPR176* in tumors, while the expression of *CHRM2* was decreased (Figure 1E). Hence, we selected *GPR176* for further research. *GPR176* expression in 249 pairs of CRC samples tended to increase in tumor tissues comparing with adjacent or normal tissues (Figure 1F). Immunohistochemistry (IHC) confirmed the upregulation of GPR176 in tumors (Figure 1G). A Chi‐square analysis showed that *GPR176* levels were correlated with tumor size and T grade, while no correlation was observed with age, gender, pathologic type lymph node metastasis, distant metastasis or primary tumor site (Table S1, Supporting Information). Kaplan–Meier analysis revealed that *GPR176* levels were associated with overall survival (HR = 2.68, *p* = 0.023) (Figure S1A, Supporting Information). These results indicated that GPR176 was upregulated and positively correlated with the poor prognosis of CRC.

### 2.2 GPR176 Promotes CRC Cell Proliferation In Vitro

**Figure 2:**
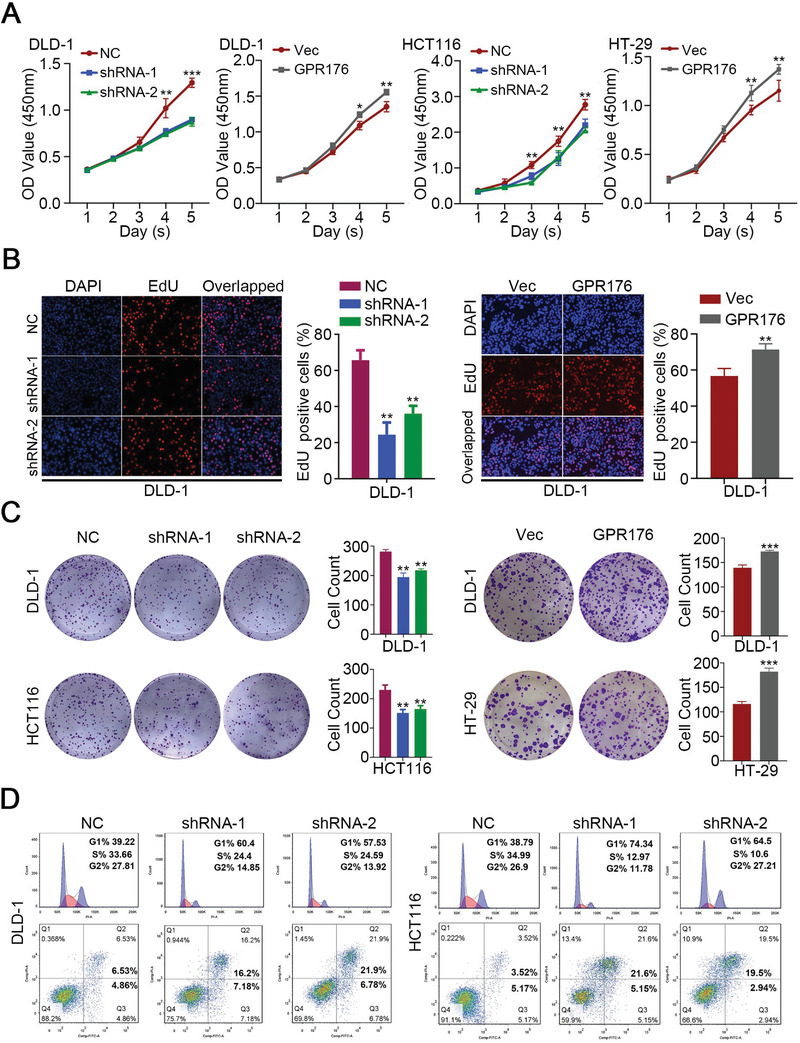
GPR176 promoted cell proliferation with the S phase arrest in vitro. A) CCK‐8 assay was performed to determine the proliferation rate of CRC cells with indicated treatment. B) Statistical results of EdU assay in CRC cells with indicated treatment. C) Statistical results of plate colony in CRC cells with indicated treatment. D) The flow cytometry (FCM) analysis of cell cycle and apoptosis in GPR176 knockdown of DLD‐1 and HCT116 cells. Data was presented with mean ± SD, ***p < 0.001, **p < 0.01 and *p < 0.05.

To detect the function of GPR176 in vitro, CCK‐8, EdU, and plate colony experiments were performed on GPR176‐knockdown (GPR176‐KD) and GPR176 overexpression (GPR176‐OE) cells. According to the expression pattern of *GPR176* in CRC cells (Figure S1B, Supporting Information), GPR176‐KD and GPR176‐OE cells were stably constructed in CRC cell lines (Figure S1C,D, Supporting Information). We observed that GPR176‐KD inhibited cell proliferation in both DLD‐1 and HCT116 cells, while GPR176‐OE promoted it (**Figure** **2**A–D, Figure S2A, Supporting Information). Flow cytometry indicated that GPR176 depletion caused G1 phase accumulation, S phase arrest, and higher apoptosis (Figure 2D, Figure S2B,C, Supporting Information). These results indicated that GPR176 stimulates cell proliferation in vitro.

### 2.3 GPR176 Enhances Tumor Development In Vivo

**Figure 3:**
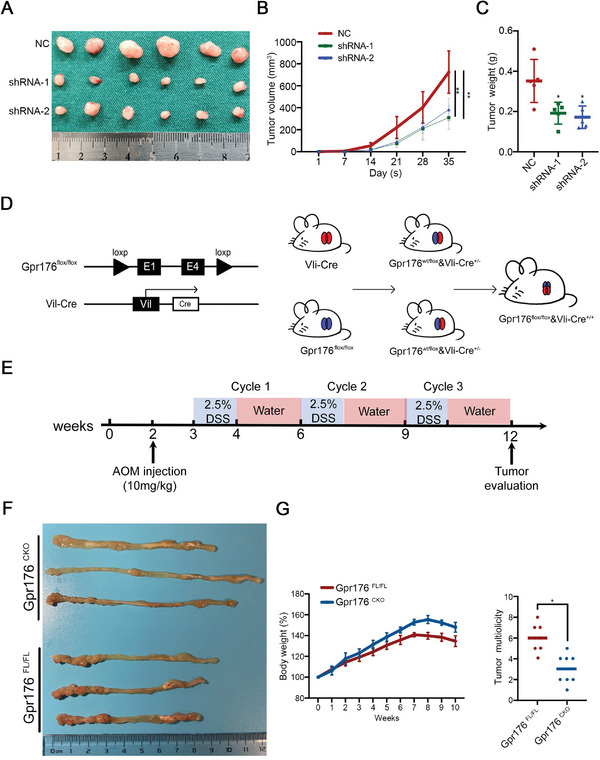
GPR176 facilitates tumor development in vivo. A) Gross evaluation of tumors in GPR176 knockdown cells in vivo on day 35 post subcutaneous injection (*n*  =  6 for each group). B,C) Tumor volume (B) and tumor weight (C in Gpr176 knockdown cells in vivo on day 35 post subcutaneous injection. D) The schematic diagram of the construction of Gpr176 CKO mice in intestine cells. E) Schematic overview of the CRC induction model. C57BL/6J mice (*n*  =  8 for each group) were injected with azoxymethane (AOM) to knock out Gpr176 in intestinal epithelial cells followed by three cycles of treatment with dextran sodium sulfate (DSS). F) Representative macroscopic images of colon tumor in Gpr176^CKO^ and Gpr176^FL/FL^ mice. G) Number of tumor per colon (left) and tumor volume (right) in AOM/DSS treated mice. Data was presented with mean ± SD, **p < 0.01 and *p < 0.05.

Another tumor model was established by subcutaneous inoculation to assess the function of GPR176 in vivo, and the results showed that GPR176‐KD significantly inhibited tumor growth (**Figure** **3**A–C). To determine whether GPR176 promotes tumor development, we constructed the Gpr176 conditional knockout (Gpr176^−CKO^) mice in the large intestine (Figure 3D), the azoxymethane and dextran sodium sulfate (AOM‐DSS) mice model was further established. The Gpr176^−CKO^ and WT Gpr176^FL/FL^ mice were fed with AOM‐DSS (Figure 3E). Gpr176^−CKO^ mice had fewer tumors and less body weight loss than WT Gpr176^FL/FL^ mice (Figure 3F,G). These results suggested that GPR176 participates in the oncogenesis and tumor development of CRC in vivo.

### 2.4 GPR176 Regulated Mitophagy in CRC

**Figure 4:**
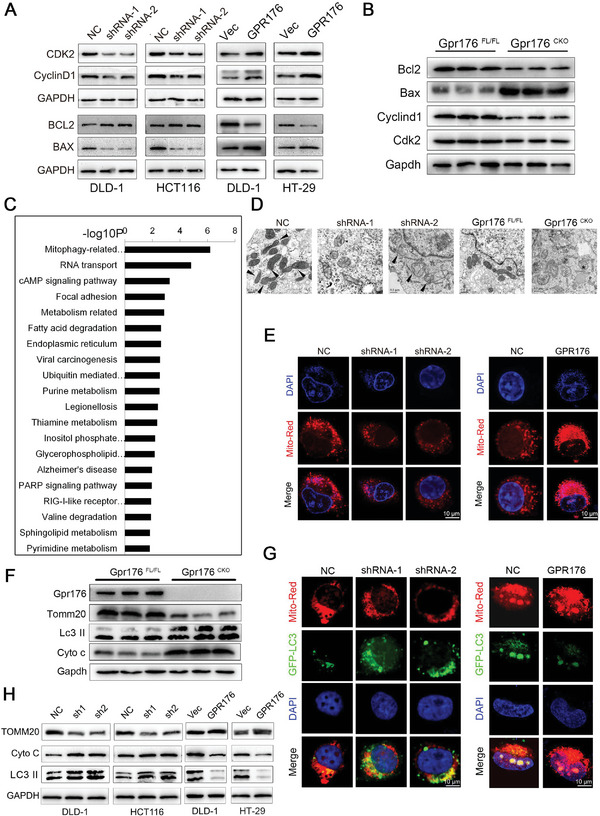
GPR176 suppressed the mitophagy in CRC cells. A) Key proteins involving in cell cycle and apoptosis were assessed by western blot in cells treated with GPR176 ovexpression or knockdown lentivirus. B) Cell cycle and apoptosis‐associated protein expression in Gpr176 CKO mice. C) Pathway enrichment analysis from the DEGs highly corrected with GPR176 expression from high throughput screening. D) The conditions of mitochondria in CRC cell lines and AOM/DSS mice model assessed by electron microscopy (EM). E) Representative images of MitoTracker Red assay in GPR176 knockdown (left) and overexpression cells (right). F) Western blot of mitochondrial membrane proteins in GPR176 CKO epithelial cells from AOM/DSS mice model. G) Immunofluorescence analysis of LC3B in GPR176 knockdown (left) and overexpression cells (right). H) Western blot of mitophagy proteins in GPR176 knockdown cells. Original magnification, ×64, bar = 10 µm (E, G), ×100, bar = 0.5 µm (D).

Western blot analysis of GPR176‐KD cells and GPR176‐OE cells, as well as tumor tissues from Gpr176^−CKO^ and WT Gpr176^FL/FL^ mice, showed significant differences in the levels of key molecules involved the cell cycle and apoptosis (**Figure** **4**A,B). Through bulk RNA‐seq of GPR176‐KD cells and control cells, DEGs were identified to assess the mechanisms by which GPR176 mediates CRC oncogenesis and development, most of which were enriched in mitophagy‐related pathways according to Gene Ontology (GO) analysis (Figure 4C). The condition of mitochondria in CRC cells from the AOM/DSS mice model and subcutaneous tumors was assessed using electron microscopy. WT Gpr176^FL/FL^ mice showed swollen mitochondria and significantly reduced mitochondrial fraction compared with Gpr176^−CKO^ mice, and these findings were supported by electron microscopy of GPR176‐KD cells in vitro (Figure 4D), suggesting mitochondrial dysfunction in these cells. According to MitoTracker Red analysis, GPR176‐KD cells showed increased mitochondrial membrane potential compared to normal controls, whereas upregulated GPR176 had the opposite effect (Figure 4E). We then probed the abundance of mitochondrial content in CKO mice in vivo, including the intermembrane protein Cyto c, outer membrane Tomm20 and autophagy monitoring protein Lc3 II. Immunoblot results indicated significantly lower Tomm20 expression but higher Cyto c and Lc3 II levels in Gpr176^−CKO^ tissues (Figure 4F). Given that LC3B initiates mitophagy in mammalian cells, CRC cells were stained with an LC3B antibody in vitro. Confocal immunofluorescence analysis revealed weaker TOMM20 and stronger LC3B signals in GPR176‐KD cells, which were reversed in GPR176‐OE cells (Figure 4G). Similarly, immunoblotting showed that the loss of GPR176 in DLD‐1 and HCT116 cells downregulated TOMM20, while increased Cyto C and LC3 II, and GPR176‐OE reversed these effects (Figure 4H). Liensinine was used to block mitophagy in CRC cells (Figure S3A, Supporting Information), and GPR176‐KD induced cell cycle arrest and cell apoptosis, and inhibited cell proliferation, while liensinine reversed these effects. (Figure S3B–S3D, Supporting Information). Similar results were obtained in the mice fed with liensinine in vivo (Figure S3E, Supporting Information). Western blot analysis of the tumor tissues from the mice showed the levels of key molecules involved the cell proliferation and apoptosis (Figure S3F, Supporting Information). Taken together, our data indicate that GPR176 upregulation impairs mitophagy, which is the primary way in which GPR176 promotes CRC progression.

### 2.5 cAMP/PKA Pathway Impaired Mitophagy In Vitro

**Figure 5:**
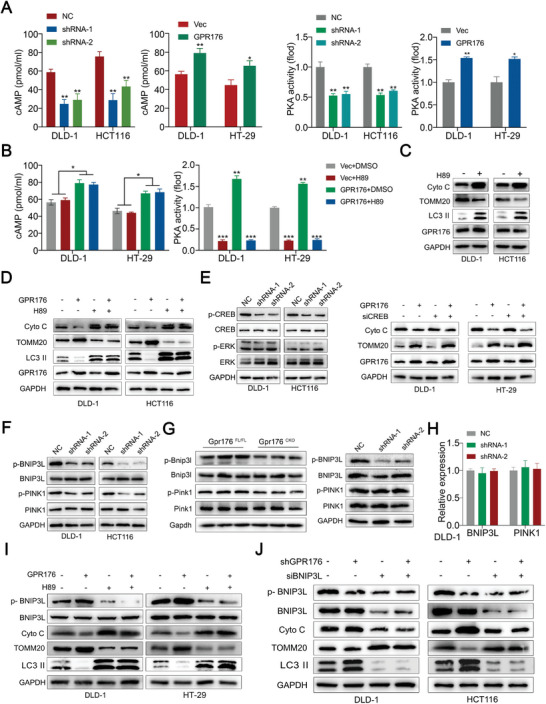
GPR176 induced cAMP/PKA activation participating in mitophagy in CRC. A) ELISA assay determined the cAMP and PKA activity in GPR176 knockdown and overexpression cells. B) The cAMP and PKA activity in GPR176 overexpressed cells treated with PKA inhibitor H89. C) Western blot of mitophagy proteins in CRC cells treated with H89. D) Western blot of mitophagy proteins in GPR176 overexpressed cells treated with PKA inhibitor H89. E) The phosphorylation of CREB and ERK in GPR176 knockdown cells or GPR176 overexpressed cells transfected with siCREB. F) The phosphorylation of mitophagy regulators (BNIP3L and PINK1) in GPR176 knockdown cells. G) Western blot of mitophagy regulators (Bnip3l and Pink1) from in vivo model (AOM/DSS‐induced mice model and subcutaneous tumors, left) and in GPR176 knockdown cells. H) Relative mRNA expression of BNIP3L and PINK1 in GPR176 knockdown cells (right). I) Western blot of BNIP3L and phosphorylation of BNIP3L in GPR176 overexpressed cells treated with H89. J) Western blot of BNIP3L and phosphorylation of BNIP3L in GPR176 knockdown cells transfected with siBNIP3L. Data was presented with mean ± SD, **p < 0.01 and *p < 0.05.

The cAMP signaling pathway was enriched in the Gene Set Enrichment Analysis (GSEA) of RNA‐seq data from GPR176‐KD cells (Figure 4C) and may be involved in the canonical signal transduction of GPCRs. Furthermore, enzyme‐linked immunosorbent assay demonstrated that cAMP levels were significantly decreased by GPR176 depletion but increased by GPR176 upregulation, and not surprisingly, PKA activity changed consistently with cAMP levels (**Figure** **5**A). We introduced H89, a potent PKA inhibitor, to pharmacologically block cAMP/PKA activation in CRC cells (Figure S4A,B, Supporting Information). H89 consistently attenuated the activation of the cAMP/PKA pathway in GPR176‐OE cells (Figure 5B) and inhibited cell proliferation (Figure S4C, Supporting Information). Given that the regulatory role of GPR176 relies on the cAMP/PKA pathway, we hypothesized that this pathway is involved in the mitophagy process. Consistent with this hypothesis, H89 enhanced Cyto C and LC3 II levels but reduced TOMM20 expression without affecting GPR176 expression (Figure 5C). Additionally, the pivotal role of GPR176 in mitophagy was abolished in the presence of H89 (Figure 5D). To assess the downstream targets of PKA, cAMP‐response element binding (CREB) protein and MAPK/ERK were investigated. In GPR176‐KD cells, we observed suppressed phosphorylation of CREB but not phosphorylation of ERK, and the lost of CREB in GPR176‐OE cells did not significantly change mitophagy protein expression (Figure 5E). Further validation of samples in vivo showed similar results (Figure S4D, Supporting Information). Similarly, CREB inhibition could not reverse the impairment of mitophagy by the cAMP analog in vitro (Figure S4E, Supporting Information), indicating that phosphorylated CREB might not be the main factor for impaired mitophagy in CRC.

### 2.6 BNIP3L Phosphorylation by cAMP/PKA Abrogated its Ability to Induce Mitophagy

Indeed, many mitochondrial proteins have been predicted to be downstream of PKA, among which some have been specifically investigated. We tested the phosphorylation status of reported mitophagy mediators, including DNMIL1, IMMT, MIC19, PTEN‐induced kinase 1 (PINK1), PRKN, and BNIP3L, in the context of GPR176 alterations. Immunoblot analysis indicated that loss of GPR176 significantly decreased the phosphorylation level of BNIP3L and PINK1 in CRC cells in vitro (Figure 5F), but not in other candidates (Figure S4F, Supporting Information). Subsequently, in vivo validation was performed. In both Gpr176^−CKO^ mice model and the subcutaneous tumor model, loss of GPR176 significantly diminished BNIP3L phosphorylation, while a slight alteration in PINK1 phosphorylation level was observed, suggesting that GPR176 mainly modulated BNIP3L phosphorylation both in vitro and in vivo (Figure 5G). Consistent with these findings, GPR176 did not alter the mRNA levels of BNIP3L and PINK1 in CRC cells (Figure 5H, Figure S4G, Supporting Information). Moreover, H89 inhibited BNIP3L phosphorylation and reduced TOMM20 while increasing Cyto C and LC3 II levels, which is regarded as an enhanced mitophagy process, especially in the presence of GPR176 (Figure 5I). We then blocked the expression of BNIP3L in CRC cells (Figure S4H, Supporting Information) and observed halted mitophagy (Figure 5J). A rescued effect was obtained when BNIP3L was re‐expressed in GPR176‐KD cells (Figure S4I,J, Supporting Information). Collectively, our data suggested that BNIP3L phosphorylation by cAMP/PKA abrogated its ability to induce mitophagy in CRC cells.

### 2.7 GPR176 Recruited GNAS Intracellularly with its Transmembrane Helix 3‐Intracellular Loop 2 Domain

**Figure 6:**
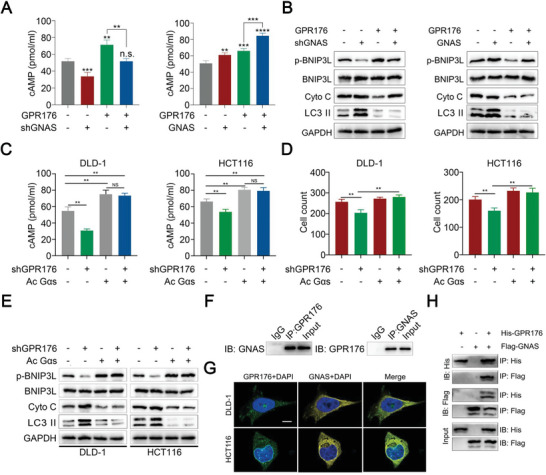
The recruitment of GNAS intracellularly is required for the function of GPR176.| A) cAMP level in cells treated with GPR176 and GNAS shRNA or overexpression lentivirus. B) Western blot of BNIP3L phosphorylation, Cyto, C, and LC3 II level in GPR176 overexpressed cells transfected with shGNAS or GNAS plasmid. C) cAMP level in GPR176 knockdown cells treated with constitutively active G*α*s. D) proliferation analysis. E) western blot of Cyto C, LC3 II, and BNIP3L phosphorylation level. F) Co‐IP assay confirmed the binding of GPR176 and GNAS. G) Colocalization images of GPR176 and GNAS determined by immunofluorescence. H) Co‐IP assay determined the binding of exogenous His‐GPR176 and FLAG‐GNAS in 293T cells. Original magnification, ×64, bar = 10 µm G) Data was presented with mean ± SD, **p < 0.01, and *p < 0.05, ns indicated no significance.

**Figure 7:**
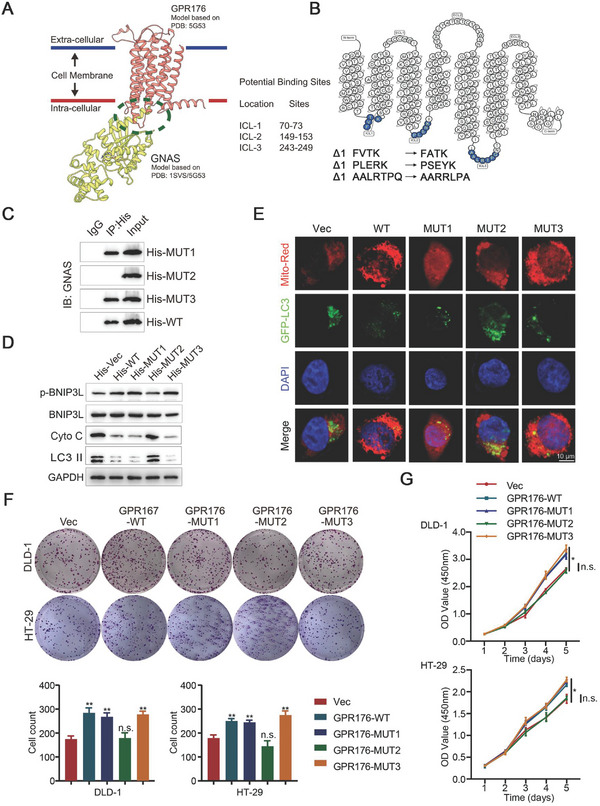
GPR176/GNAS complex maintains the proliferation of CRC. A) The interaction of GPR176/GNAS predicted by homology modeling. B) Three mutants of potential GNAS binding regions in GPR176 were shown in blue. C) Co‐IP assay confirmed the potential GNAS binding regions in GPR176. D) western blot of Cyto C, LC3 II, and BNIP3L phosphorylation level. E) Immunofluorescence analysis of LC3B in three mutants of potential GNAS binding regions in cell lines. F) Plate colony assay in cells treated with three mutant GPR176. G) CCK‐8 in cells treated with three mutant GPR176. Original magnification, ×64, bar = 10 µm (E), Data was presented with mean ± SD, **p < 0.01, and *p < 0.05, ns indicated no significance.

The elevation of cAMP in CRC might be partially attributed to the interaction of GPR176 with G*α* subunits. Therefore, we investigated whether the binding of GPR176 to GNAS activated the cAMP/PKA pathway. Knockdown of GNAS attenuated the induction of cAMP by GPR176, whereas forced GNAS exhibited the opposite effect in CRC cells (**Figure** **6**A; Figure S5A,B, Supporting Information). The GPR176‐regulated‐cAMP/PKA pathway was involved in mitophagy, and immunoblot analysis confirmed that GNAS controlled BNIP3L phosphorylation, Cyto C, and LC3 II expression in vitro (Figure 6B). Moreover, introducing constitutively active G*α*s (Ac G*α*s) prevented GPR176‐KD‐induced inhibition of cAMP, proliferation arrest, and mitophagy onset in CRC cells (Figure 6C–E). These results suggested that GNAS participates in the GPR176‐cAMP/PKA‐BNIP3L pathway, which blocked mitophagy in CRC cells. A Co‐IP assay confirmed the interaction between GPR176 and GNAS in CRC cells (Figure 6F, Figure S5C, Supporting Information). Immunofluorescence (IF) colocalization analysis indicated a fair uniformity of protein distribution in CRC cells (Figure 6G). Consistently, co‐IP of His‐tagged GPR176 with FLAG‐tagged GNAS in 293T cells showed the same results (Figure 6H). Putative binding sites between GPR176 and GNAS were assessed. Using an advanced homology modeling tool, the 3D structure of the GPR176 transmembrane domain and GNAS domain was constructed. Based on the homology model, we predicted that the intracellular domain of GPR176 bound to GNAS is located in the intracellular loop (ICL) 1 (70–73), transmembrane helix (TMH) 3‐ICL2 (149–153), and ICL3‐TMH6 (243–249) (**Figure** **7**A). To further confirm the precise site, three mutant plasmids with GPR176 deleted potential binding domain were generated (Figure 7B). Co‐IP results indicated that only mutant 2 showed little or no binding capacity with GNAS compared to its counterparts (Figure 7C), demonstrating that TMH3‐ICL2 (149–153) was the binding domain of GPR176 with GNAS. We investigated the necessity of the TMH3‐ICL2 domain for GPR176‐mediated mitophagy in CRC cells. We observed that GPR176 (GPR176‐WT), GPR176‐MUT1, and GPR176‐MUT3, but not GPR176‐MUT2, impaired mitophagy (Figure 7D,E). These results suggest that GPR176 recruits GNAS intracellularly to block mitophagy in CRC cells.

### 2.8 GPR176/GNAS Complex Regulated Cell Proliferation in CRC

A proliferation assay, to further investigate the function of the GPR176/GNAS complex in CRC, showed that GPR176 wild‐type (GPR176‐WT) plasmid, GPR176‐MUT1 plasmid, and GPR176‐MUT3 plasmid, but not GPR176‐MUT2 plasmid, significantly enhanced cell proliferation (Figure 7F,G). Moreover, in GPR176‐OE cells, sh*GNAS* abolished the stimulatory role of GPR176 (Figure S5D,E, Supporting Information). A xenograft model was used to understand the GPR176/GNAS complex in vivo. Four groups of mice were injected with differentially pretreated CRC cells: GPR176 vector control (*V*ec), GPR176 overexpression (GPR176), GPR176‐OE combined with GNAS overexpression (GPR176+*GNAS*), and GPR176 overexpression combined with *GNAS* knockdown (GPR176+sh*GNAS*). We observed that GPR176‐OE promoted the proliferation of CRC cells, compared to the normal control, while GNAS knockdown nullified the stimulatory effects of GPR176 (Figure S5F–S5J, Supporting Information). Additionally, siRNA targeting the 3″‐UTR of GPR176 was used to block GPR176 in CRC cells. Neither shGPR176 nor siGPR176‐3″UTR affected GNAS mRNA level and protein level (Figure S6A,B). Overexpression of GNAS inhibited mitophagy with the involvement of GPR176‐WT but not GPR176‐Mut2 (Figure S6C). After blocked endogenous GPR176, GNAS‐OE could not rescue cell cycle arrest, increased apoptosis, and reduced cell proliferation, unless exogenous GPR176‐WT but not GPR176‐Mut2 was involved (Figure S6D–S6G, Supporting Information). These results suggest that GPR176 promotes CRC progression by interacting with GNAS in vitro and in vivo.

## 3 Discussion

Dysregulation of GPCRs accelerates tumorigenesis and metastasis, modulates cancer cells themselves, and spatiotemporally controls immune cells. For example, GPR68, as a proton‐sensing GPCR, mediates the interaction between cancer‐associated fibroblasts and cancer cells, thereby promoting cell proliferation in pancreatic ductal adenocarcinoma.^[^ ^16^ ^]^ Similarly, Song et al. found that JTC801 induces cell death through the GPCR pathway by reducing the expression of CA9.^[^ ^17^ ^]^ Therefore, GPCRs are an attractive research target for cancer diagnosis, treatment, and prevention. Several studies have focused on GPCRs in patients with CRC. GPR15 modified regulatory T‐cell‐guided antitumor immunity, promoted intestinal tumorigenesis, and regulated the tumor microenvironment.^[^ ^18^ ^]^ Moreover, CCK2R, known for its transporting role in cholecystokinin, regulated progastrin‐dependent tumor development in CRC.^[^ ^19^ ^]^ We previously determined the role of GPR56 in promoting cell proliferation in CRC via the PI3K/AKT signaling pathway.^[^ ^20^ ^]^ To further our understanding of GPCRs in CRC, we used bulk screening and identified an orphan GPCR, GPR176, which was overexpressed and highly correlated with poor prognosis in CRC. Both the mice model and functional assays demonstrated its protumorigenic effects in CRC.

Since the underlying mechanism remains unclear, a series of experiments defined key events at the transcriptional level and mitophagy was also enriched. Based on electron micrographs, a reduced fraction and swollen mitochondria appeared. TOMM20 and Cyto C are mitochondrial membrane proteins that represent mitochondrial content and could be regulated by mitophagy.^[^ ^21^ ^]^ Immunofluorescence revealed that the levels of these proteins were closely associated with GPR176 in CRC cells but not normal cells (Figure S7A, Supporting Information). Meanwhile, the overexpression of GPR176 was specific in CRC (Figure S7B, Supporting Information). In CRC cells, multiple pathways determine mitophagy. cAMP signaling pathway was previously identified as a regulator of mitochondrial dynamics.^[^ ^22^ ^]^ We found that either H89 or a cAMP analog affected mitophagy in CRC cells. Additionally, cAMP levels and PKA activity were positively correlated with GPR176, leading to the inhibition of mitophagy. Many proteins are the downstream targets and phosphorylated by cAMP/PKA, such as CREB and Raf. ^[^ ^23^ ^]^. CREB, as the main substrate of PKA, stimulates the transcription of certain genes, which further participate in mitophagy.^[^ ^24^ ^]^ Additionally, the MAPK/ERK pathway maintains proper mitochondrial functions.^[^ ^25^ ^]^ Although our results showed that CREB, but not Raf, was regulated by GPR176‐mediated cAMP/PKA, but further validation confirmed that CREB might not be involved in GPR176‐mediated mitophagy, suggesting that PKA‐phosphorylated CREB was not the primary factor for impaired mitophagy in CRC. Considering the importance of CREB to cancer progression, other mechanisms should exist, and further research is required to reveal them. BNIP3L met the requirements of a PKA substrate.^[^ ^26^ ^]^ First, the phosphorylation level of BNIP3L was reduced in GPR176^−CKO^ mice and the subcutaneous tumor model but was enhanced in tumor tissues. Second, alteration of GPR176 expression or cAMP/PKA activity affected BNIP3L phosphorylation in vitro. Third, mutation of BNIP3L phosphorylation site by PKA attenuated its regulatory role in mitophagy. All these indicated that BNIP3L is the main prerequisite for GPR176‐mediated mitophagy in CRC. Although PINK1 was also affected by the lost of GPR176, we did not observe significant changes in phosphorylation levels, especially in vivo, potentially due to existing in vivo factors that counterbalanced PKA phosphorylation.

GPCRs rely mainly on the interaction of G proteins to exert their functions intracellularly. The *α*, *β*, and *γ* subunits constitute heterotrimeric G proteins.^[^ ^27^ ^]^ The G*α* subunit plays a key role in guiding the activation and termination of GPCR signaling.^[^ ^28^ ^]^ We identified *GNAS* empirically, and in vitro/vivo validation verified the important role of GNAS in GPR176‐mediated mitophagy. Through homology modeling, we determined the precise binding domain of GPR176 with GNAS, uncovering the mechanisms of the GPR176/ GNAS axis in mitophagy control. Thus, we confirmed that GNAS transmitted signals from GPR176 to activate cAMP/PKA/BNIP3L, resulting in CRC cell survival and proliferation. In addition to G proteins, G‐protein signal transduction regulator proteins (RGSs) act as scaffolds to assemble related proteins in the route of signal transduction of G proteins.^[^ ^29^ ^]^ Thus, RGSs are regarded as the downstream nodes of GPCRs. However, whether GPR176 GNAS activation relies on RGS guiding or is independent remains to be elucidated in future experiments.

**Figure 8:**
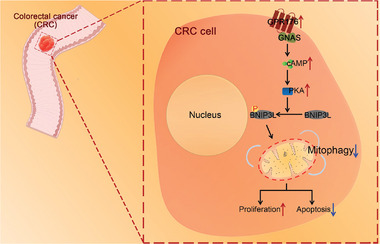
Schematic diagram of hypothesis. During CRC progression, GPR176 recruits GNAS, thereby limiting mitophagy via the cAMP/PKA/ BNIP3L axis, then promoting the tumorigenesis and progression of CRC.

In conclusion, we demonstrated that aberrant GPR176 expression is correlated with poor prognosis. GPR176 plays a key role in modulating CRC proliferation owing to its high affinity for GNAS. Mechanistically, upon binding to GNAS, GPR176 continuously activates the cAMP/PKA/BNIP3L cascade and further abolishes the activation of mitophagy, causing the occurrence and development of CRC (**Figure** **8**). Our findings may provide novel insights for the early diagnosis of CRC and a rational drug target to treat patients with CRC.

## 4 Experimental Section

### Generation of GPR176*^−^* ^CKO^ Mice

GPR176 conditional knockout mice (GPR176^−CKO^) with the deletion of *GPR176* exons 1–4 in intestinal cells were constructed using Cre recombinase expressed by the intestinal‐cell‐specific villin 1 (Vil1) promote. The GPR176 ^FL/FL^ mice were first generated by breeding the floxed allele into homozygosity and then crossed with Vil1‐Cre mice to obtain GPR176^−CKO^ mice. All the mice were on the C57BL/6 background.

### Animal Models

All animal experiments were approved by the Animal Care and Use Committee of Nanjing Medical University and were performed according to the guidelines of the National Institutes of Health (IACUC‐2212011). For the AOM/DSS model, female C57BL6 mice (8 weeks of age) were maintained at the Animal Core Facility of Nanjing Medical University. Briefly, GPR176^−CKO^ or GPR176 ^FL/FL^ mice were injected intraperitoneally with AOM (10 mg kg^−1^ body weight) (Sigma–Aldrich). After 1 week of AOM administration, the mice received 2% (w/v) DSS (MP Biochemicals) in drinking water for 5 days, followed by a rest period without DSS for 2 weeks. This 5‐day cycle was repeated twice. All mice were sacrificed on day 84, and the colon was excised and flushed with PBS. Subsequently, the colon was inspected, photographed, and stained with hematoxylin and eosin. The body weight of the mice was recorded over time from day one. For the tumor‐bearing model, 5‐week‐old male BALB/c nude mice were used. Briefly, CRC cells stably transfected with certain shRNAs, or normal controls were injected subcutaneously into the right or left flank of the mice. Tumor growth was monitored every 3 days. All mice were sacrificed ≈5 weeks later, and the tumors were dissected and embedded in paraffin for hematoxylin and eosin, and IHC staining.

Additional details are provided in Supplementary Materials.

## Conflict of Interest

The authors declare no conflict of interest.

## Author Contributions

J.T., W.P., and J.J. contributed equally to this work. Y.M.S conceived the project and supervised all experiments. J.W.T, W.P, J.Z.J, C.F.P., and J.O.G. conducted all experiments and analyzed the data. K.P.J and X.W.W were responsible for clinical sample collection. K.P.J, X.W.W, T.W, C.F.P., and P.Y provided support with experimental techniques. J.W.T, Y.F.F., and T.W. constructed the manuscript. All authors read and approved the final manuscript.

## Supporting information

Supporting Information

## Data Availability

The data that support the findings of this study are available from the corresponding author upon reasonable request.
