# Supplementary material for: GPR176 Promotes Cancer Progression by Interacting with G Protein GNAS to Restrain Cell Mitophagy in Colorectal Cancer
Source: Adv Sci (Weinh). 2023 Mar 11;10(12):2205627. doi: 10.1002/advs.202205627 (PMC10131842; doi:10.1002/advs.202205627)
Supplement: Supplementary file 1 — Supporting Information [file ADVS-10-2205627-s001.pdf]

ADVANCED SCIENCE
Open Access

Supporting Information

for *Adv. Sci.*, DOI 10.1002/advs.202205627

GPR176 Promotes Cancer Progression by Interacting with G Protein GNAS to Restrain Cell Mitophagy in Colorectal Cancer

Junwei Tang, Wen Peng, Jiangzhou Ji, Chaofan Peng, Tuo Wang, Peng Yang, Ji'ou Gu, Yifei Feng, Kangpeng Jin, Xiaowei Wang* and Yueming Sun**

Supplementary Materials

GPR176 promotes cancer progression by interacting with G protein GNAS to restrain cell mitophagy in colorectal cancer

Junwei Tang[1, 2,#], Wen Peng[1, 2,#], Jiangzhou Ji[1, 2, 3,#], Chaofan Peng[1,2,3], Tuo Wang[1, 2, 3], Peng Yang[1, 2, 3], Ji'ou Gu[1, 2], Yifei Feng[1, 2], Kangpeng Jin[1, 2,*], Xiaowei Wang[1, 2, 3,*], Yueming Sun[1, 2,*]

[1] Department of General Surgery, The First Affiliated Hospital of Nanjing Medical University, Nanjing Jiangsu, 210029, China; [2] Colorectal Institute of Nanjing Medical University, Nanjing, China; [3] The First School of Clinical Medicine, Nanjing Medical University, Nanjing, China

[#]Equal contribution

[*]Corresponding authors

This file includes:

Supplementary Methods

Supplementary Table 1-2

Supplementary Figure legends

Supplementary Figure 1-7

Contract information

Dr. Yueming Sun

E-mail address: sunyueming@njmu.edu.cn

Department of General Surgery, The First Affiliated Hospital of Nanjing Medical University, Colorectal Institute of Nanjing Medical University, Nanjing, Jiangsu, 210029, China. Phone: +862568306026; Fax: +862568306026

Supplementary Methods

Patient samples

After providing informed consent, tissue samples were collected from patients who underwent surgery at the First Affiliated Hospital of Nanjing Medical University between 2009 and 2010. All experiments were performed in compliance with government policies and the Declaration of Helsinki. This study was approved by the human ethics committee of the First Affiliated Hospital of Nanjing Medical University.

Cell proliferation and migration assay

Cell counting Kit-8 (CCK-8, Dojindo, Japan), EdU (ab222421, Abcam, US), and plate colony assays were used to quantify the growth of the CRC cells. Corning Transwell (8.0 um pore polycarbonate membrane insert, Cat#3422) was used to evaluate cell migration. All procedures were performed following the manufacturer's protocols.

Cell cycle and apoptosis analysis

For the cell cycle assay, pretreated cells were stained with propidium iodide (PI) (BD Biosciences), according to the manufacturer's instructions. For the apoptosis assay, pretreated cells were collected by centrifugation and resuspended in 500 µL 1X binding buffer. Then 5 µL of annexin V-FITC and 5 µL of PI were added and incubated in the dark for 5 minutes. Finally, the percentage of apoptotic cells was quantified by flow cytometry.

RNA-seq and enrichment analysis

Total RNA was extracted from tumor tissues and cancer cells using TRIzol reagent (Thermo Fisher Scientific Inc., Waltham, MA, USA). RNA-seq and data analysis were performed by CapitalBio Technology (Beijing, China). GO enrichment analysis, GSEA and visualization of DEGs were performed using the cluster Profiler R package[1]. Genes with adjusted P-values < 0.05 were considered significantly enriched by DEGs.

Cell culture

Human CRC cell lines (SW480,SW620, RKO, HT29, DLD-1, HCT116, and LoVo) and normal epithelial cells of the colon (FHC) were obtained from the Cell Bank of Type Culture Collection of the Chinese Academy of Sciences (Shanghai, China). All cells were cultured in their corresponding medium, incubated in a 5% CO_2 humidified incubator at 37°C.

sh/siRNAs and lentivirus production

For transient transfection, Lipofectamine 3000 and Opti-MEM (Invitrogen) were used, according to the manufacturer's instructions. The lentivirus used for the stable knockdown or overexpression of genes was designed and purchased from OBiO Technology (Shanghai, China). All sequences are listed in **Supplementary Table 2**.

RNA preparation and qRT-PCR

Total RNA of cells and tissues was obtained and then reverse transcribed into cDNA using the PrimeScript RT reagent kit (TaKara, Dalian, China). The SYBR Premix Ex Taq Kit (TaKaRa) was used to perform qRT-PCR on an Applied Biosystems 7500 Sequence Detection System (Applied Biosystems). Primer sequences for qRT-PCR are listed in **Supplementary Table 2**.

Western blot and immunohistochemistry

For western blot assay, protein lysates were extracted from tissues and cultured cells using RIPA buffer (Beyotime, China). A BCA kit (Beyotime) was used to quantify the protein concentration. Equal amounts of protein were separated using SDS-PAGE. For IHC, tumor samples were embedded in paraffin and sectioned into 4 mm slices. The slices were first incubated with primary antibodies against the target antigens. Secondary antibodies were used to bind to the primary antibody and detected using the detection system. Antibodies against phospho-IMMT (Ser528) and phospho-MIC19 (Thr11) were generated using the synthetic phospho-peptides[2]. All antibodies used in this experiment are listed in (**supplementary table 2**).

Generation of GPR176^{-CKO} mice

GPR176 conditional knockout mice (GPR176^{-CKO}) with the deletion of *GPR176* **exons 1–4** in intestinal cells were constructed using Cre recombinase expressed by the intestinal-cell-specific villin 1 (Vil1) promote. The GPR176 $^{FL/FL}$ mice were first

generated by breeding the floxed allele into homozygosity and then crossed with Vil1-Cre mice to obtain GPR176^{-CKO} mice. All the mice were on the C57BL/6 background.

Animal models

All animal experiments were approved by the Animal Care and Use Committee of Nanjing Medical University and were performed according to the guidelines of the National Institutes of Health. For the AOM/DSS model, female C57BL6 mice (8 weeks of age) were maintained at the Animal Core Facility of Nanjing Medical University. Briefly, GPR176^{-CKO} or GPR176 $^{FL/FL}$ mice were injected intraperitoneally with AOM (10 mg/kg body weight) (Sigma-Aldrich). After 1 week of AOM administration, the mice received 2% (w/v) DSS (MP Biochemicals) in drinking water for 5 days, followed by a rest period without DSS for 2 weeks. This 5-day cycle was repeated twice. All mice were sacrificed on day 84, and the colon was excised and flushed with PBS. Subsequently, the colon was inspected, photographed, and stained with hematoxylin and eosin. The body weight of the mice was recorded over time from day one. For the tumor-bearing model, 5-week-old male BALB/c nude mice were used. Briefly, CRC cells stably transfected with certain shRNAs, or normal controls were injected subcutaneously into the right or left flank of the mice. Tumor growth was monitored every 3 days. All mice were sacrificed approximately 5 weeks later, and the tumors were dissected and embedded in paraffin for hematoxylin and eosin and IHC staining. The mice described in Supplementary Figure S3E were fed with liensinine (10 mg/kg/day in drinking water)[3] (Pureone Bio Technology; #P0943)

Electron microscopy

Samples, including tumor tissues and CRC cells, were fixed in 4% formaldehyde and 1% glutaraldehyde. They were then processed for transmission electron microscopy using a Hitachi H-500 electron microscope (FEI, USA).

MitoTracker Red

To assess the mitochondrial membrane potential in CRC cells, a MitoTracker Red kit (Invitrogen) was used (final concentration 100 nM). Briefly, cells were grown on coverslips inside a Petri dish. When the cells reached the desired confluency, they

were fixed with growth medium containing 3% formaldehyde at 37°C for 15 min. After fixation, the cells were rinsed three times in PBS and stained with a solution containing MitoTracker Red for 20 min at 37°C. All cells were then observed under a fluorescence microscope (Leica Microsystems CMS Gmbh Ernst-Leitz-Str, 17-37) and analyzed using Leica Application Suite X, 3.6.20104.0.

Enzyme-linked immunosorbent assay

Human cAMP enzyme-linked immunosorbent assay kits (E-EL-0056c) were purchased from ElabScience (Wuhan, China). Briefly, 50 µl of the standard or test samples was added to the plate. Then, 50 µl of biotinylated antibody was immediately added to each well and incubated at 37°C for 45 min. After washing three times, 100 µl of HRP-conjugated working solution was added to each well and incubated at 37°C for 30 min, followed by the addition of 90 µl substrate solution and incubation at 37°C for 15 min. Next, the terminating solution was added, and absorbance values were read at 450 nm.

Co-immunoprecipitation assay

The Co-IP assay was performed as described previously[4]. The corresponding antibodies were used to capture the protein complexes. The precipitate was then subjected to western blotting or mass spectrum analysis.

Immunofluorescence assay (IF)

IF was performed as previously described[5]. First, the test cells were grown to the appropriate density and then washed with PBS. Fixed cells in PBS containing 10% formalin for 10 minutes. The cells were rinsed in PBS-Tween 20 for 2 × 2 min, followed by serum blocking and incubation in a primary antibody dilution buffer for 1 h at 25℃. The cells were then incubated with a secondary antibody for 20–30 minutes at 25℃. After rinsing in PBS-Tween 20 for 3 × 2 min, the procedures were performed using the second antibody. After the above steps were completed, samples were stained with DAPI and analyzed with a confocal microscope.

Homology modeling

We generated GPR176 and GNAS homology models using the Advanced Homology Modeling tool based on the Maestro platform in Schrodinger (2015. v4). The GPR176

model was based on PBD:5G53 and *GNAS* was based on 1SVS. The potential binding locations were established based on the homology models. Mutant plasmids were then constructed using OBiO Technology (Shanghai, China).

Statistical analysis

All statistical analyses were performed by using R-software (version 4.1.0) and visualized by using Prism (La Jolla, CA, USA). Chi-square test, Student's *t*-test, and Pearson's correlation analysis were used. All experiments were independently repeated at least three times. Figures were prepared using Serif Affinity Photo (Serif, UK). $P<0.05$ was considered statistically significant (*, $p<0.05$; **, $p<0.01$; ***, $p<0.001$; ****, $p<0.0001$).

References

[1] a) T. Wu, E. Hu, S. Xu, M. Chen, P. Guo, Z. Dai, T. Feng, L. Zhou, W. Tang, L. Zhan, X. Fu, S. Liu, X. Bo, G. Yu, *Innovation (Camb)* **2021**, *2* (3), 100141, https://doi.org/10.1016/j.xinn.2021.100141; b) G. Yu, L. G. Wang, Y. Han, Q. Y. He, *Omics* **2012**, *16* (5), 284, https://doi.org/10.1089/omi.2011.0118.

[2] S. Akabane, M. Uno, N. Tani, S. Shimazaki, N. Ebara, H. Kato, H. Kosako, T. Oka, *Molecular cell* **2016**, *62* (3), 371, https://doi.org/10.1016/j.molcel.2016.03.037.

[3] J. Zhou, G. Li, Y. Zheng, H. M. Shen, X. Hu, Q. L. Ming, C. Huang, P. Li, N. Gao, *Autophagy* **2015**, *11* (8), 1259, https://doi.org/10.1080/15548627.2015.1056970.

[4] L. Dan, L. Liu, Y. Sun, J. Song, Q. Yin, G. Zhang, F. Qi, Z. Hu, Z. Yang, Z. Zhou, Y. Hu, L. Zhang, J. Ji, X. Zhao, Y. Jin, M. A. McNutt, Y. Yin, *Nat Immunol* **2020**, *21* (3), 287, https://doi.org/10.1038/s41590-019-0577-9.

[5] Y. Yin, X. Xu, J. Tang, W. Zhang, G. Zhangyuan, J. Ji, L. Deng, S. Lu, H. Zhuo, B. Sun, *Hepatology (Baltimore, Md.)* **2018**, *68* (5), 1865, https://doi.org/10.1002/hep.30068.

Supplementary Tables

Supplementary Table 1. The clinicopathological characteristics of patients with CRC.

	n	GPR176 [high]	GPR176 [low]	*p* value[a]
	**n**	**n=135**	**n=134**	
**Age(years)**				
<60	122	63	59	0.664
≥60	147	72	75	
**Gender**				
Male	165	86	79	0.424
Female	104	49	55	
**Tumor diameter (cm)**				
<3cm	120	50	70	0.012
≥3cm	149	85	64	
**Pathologic type**				
ADC	140	73	67	0.504
MADC	129	62	67	
**Lymph node metastasis**				
No	124	66	58	0.418
Yes	145	69	76	
**T stage**				
T1	48	18	30	0.002
T2	79	30	49	
T3	86	54	32	
T4	55	33	23	
**Distant metastasis**				
No	52	23	29	0.339
Yes	217	112	105	
**Primary tumor site**				
Colon	157	81	76	0.585
Rectum	112	54	58	

a: Chi-square test, ADC: Adenocarcinoma; MADC: Mucinous adenocarcinoma

Supplementary Table 2

1. Primer sequences

**Name**	**Sequence (5' -> 3')**	**Supplier**
Human-CHRM2(Forward Primer)	AACTCCTCTAACAATAGCCTGGC	Realgene
Human-CHRM2 (Reverse Primer)	GTTCCCGATAATGGTCACCAAA	Realgene
Human-CHRM3(Forward Primer)	CACGTACCGAGCCAAACGAA	Realgene
Human-CHRM3(Reverse Primer)	AGGACAAAGGAGATGACCCAA	Realgene
Human-OR12D3 (Forward Primer)	TGCACACCTGAGTTTTTGTGG	Realgene
Human-OR12D3(Reverse Primer)	GGCCAATTCTAAGAGCGGCTT	Realgene
Human-GPR176(Forward Primer)	TGGACAGGTACTACTCAGTCCT	Realgene
Human-GPR176 (Reverse Primer)	TGGACGTGGCATAGATGTCAG	Realgene
Human-GPR15(Forward Primer)	TTACTATGCTACGAGCCCAAACT	Realgene
Human-GPR15(Reverse Primer)	CTCCCATGAGAACAAGGTTCC	Realgene
Human-FZD7 (Forward Primer)	GTGCCAACGGCCTGATGTA	Realgene
Human-FZD7 (Reverse Primer)	AGGTGAGAACGGTAAAGAGCG	Realgene
Human-OR52B6(Forward Primer)	GCACCTGCACTATTGCCAGAT	Realgene
Human-OR52B6(Reverse Primer)	CTGCCAACCCATACCAGACAT	Realgene
Human-DRD3(Forward Primer)	AGAAGGCAACCCAAATGGTGG	Realgene
Human-DRD3(Reverse Primer)	TGTCGTGGCACTGTAAAGCTC	Realgene
Human-OR6C75(Forward Primer)	CACAGTGGCAGGTTGTACTTT	Realgene
Human-OR6C75(Reverse Primer)	AATGCAGACAGACGTGAATGAA	Realgene
Human-OR10G8(Forward Primer)	GCGCTCGTGTACTCTTCTGG	Realgene
Human-OR10G8(Reverse Primer)	CCGAGGCCACTATTCCAACAG	Realgene
Human-SSTR1(Forward Primer)	CCAGCATCTACTGTCTGACTGT	Realgene
Human-SSTR1(Reverse Primer)	ATGACGAGCAGCGATAGCAC	Realgene
Human-GLP2R(Forward Primer)	TCCTGGAAATGTCTCTGTACCC	Realgene
Human-GLP2R(Reverse Primer)	GGCGTTCTCTATCGTCTGCC	Realgene
Human-FZD6(Forward Primer)	GCGATAGCACAGCCTGCAATA	Realgene
Human-FZD6(Reverse Primer)	AATGGTAAGAATCACCCACCAC	Realgene
Human-PTGDR(Forward Primer)	GGTGCTTTATCCAGATGGTCC	Realgene
Human-PTGDR(Reverse Primer)	GTGCATCGCATAGAGGTTGC	Realgene
Human-VIPR1(Forward Primer)	TCATCCGAATCCTGCTTCAGA	Realgene
Human-VIPR1(Reverse Primer)	AGGCGAACATGATGTAGTGTACT	Realgene
Human-OR5A2(Forward Primer)	CAATCTGCAACCCCTTGCTTT	Realgene
Human-OR5A2(Reverse Primer)	ACACTGACTATGAAGGTCACCA	Realgene
Mouse-GPR176(Forward Primer)	GGTGTTATGGTCAACTTGCCG	Realgene
Mouse-GPR176(Reverse Primer)	AGAGCATCGTATAGATCCACCAG	Realgene

2. Antibodies

Name	Supplier	Cat no.
GPR176	Abcam	ab122605
GPR176	G-Biosciences	ITT2003
BCL2	Abcam	ab182858
BAX	Proteintech	50599-2-Ig
CyclinD1	Abcam	ab16663
CDK2	Abcam	ab32147
CDK4	Abcam	ab108357
GAPDH	Cell Signaling Technology	#5174
Cyto C	Cell Signaling Technology	#11940
TIMM23	Abcam	ab230253
TOMM20	Abcam	ab186735
ATPB	Abcam	ab14730
p-CREB	Cell Signaling Technology	#9198
CREB	Cell Signaling Technology	#9197
p-ERK	Cell Signaling Technology	#4370
ERK	Cell Signaling Technology	#4695
p-BNIP3L	Abcam	ab109414
BNIP3L	Abcam	ab208190
p-PINK1	Cell Signaling Technology	#46421
PINK1	Cell Signaling Technology	#6946
p-DNM1L	Abcam	ab193216
DNM1L	Abcam	ab184247
p-IMMT	This paper	
IMMT	Abcam	ab137057
p-MIC19	This paper	
MIC19	Abcam	ab224565
p-PRKN	Cell Signaling Technology	#36728
PRKN	Cell Signaling Technology	#4211
GNAS	Abcam	ab283266
IgG	Abcam	ab172730
LC3 II	Abcam	ab51520

3. Knockdown sequences

Name	Sense	Antisense	Supplier
Human-GPR176	CCCTCCAGAACATCTCCCCAT	TATATTATCAGAACAACAC	GenePharma, Shanghai
	GGTACTACTCAGTCCTCTA	TAGAGGACTGAGTAGTACC	
	GCTCTGTCTTTCACCTTAA	TTAAGGTGAAAGACAGAGC	
3'-UTR of GPR176	GTGTTGTTCTGATAATATATT	TATATTATCAGAACAACACTT	
Human-GNAS	CTTAGATGTTCCAAATTTA	TAAATTTGGAACATCTAAG	GenePharma, Shanghai
	CTGTGATCCTGTTCCTCAA	TTGAGGAACAGGATCACAG	
	CAAAGTGCAGGACATCAAA	TTTGATGTCCTGCACTTTG	
Human-BNIP3L	GGCUCAAGAUGUUUGUAAAUA	UUUACAAACAUCUUGAGCCAG	GenePharma, Shanghai
	GAGAAUUGUUUCAGAGUUAUU	UAACUCUGAAACAAUUCUCUU	
	CAGAGUUUACUGUUGUUUAGA	UAAACAACAGUAAACUCUGUU	
Human-PKA	GCGACATCAAGAACCACAATT	TTGTGGTTCTTGATGTCGCTT	GenePharma, Shanghai

Supplementary Figure legends

Supplementary Figure 1. GPR176 predicted poor prognosis and dysregulated in CRC cells.

A, Kaplan-Meier analysis presented with high and low expression of GPR176 in patients. **B,** The mRNA and protein expression patterns of GPR176 in selected cell lines of CRC. **C,** The validation of GPR176 knockdown efficiency in DLD-1 and HCT116 cells. **D,** The validation of GPR176 overexprssion efficiency in DLD-1 and HT-29 cells. Data was presented with Mean±SD, *** $p < 0.001$ and ** $p < 0.01$.

Supplementary Figure 2. Regulation of GPR176 in the CRC cancer cells.

A, Statistical results of EdU assay in HCT116 and HT-29 cells with indicated treatment. **B-C,** Statistical results of flow cytometry analysis of cell cycle and apoptosis distribution in GPR176 knockdown cells.

Supplementary Figure 3. GPR176 affected CRC progression via inhibiting mitophagy. A, Western blot of mitophagy proteins in GPR176 knockdown cells treated with liensinine. **B-C,** Flow cytometry analysis of cell cycle and apoptosis in GPR176 knockdown cells treated with liensinine. **D,** Representative images of plate colony assay in GPR176 knockdown cells treated with liensinine. **E,** Tumor formation of GPR176 knockdown cells in liensinine fed mice (n = 3 for each group). **F,** Western blot of the key molecules involved in the cell proliferation and apotosis.

Supplementary Figure 4. BNIP3L phosphorylation by PKA impairs induction of mitophagy.

A, B, The calculation of the half-maximal inhibitory concentration (IC50) of H89 in CRC cells. **C,** CCK-8 assay in GPR176 overexpressed cells treated with H89. **D,** Western blot analysis of the phosphorylation level of CREB and ERK in GPR176 CKO mice model and GPR176 knockdown cells. **E,** Western blot of mitophagy proteins in CRC cells treated with cAMP analog (cAMP) and/or siCREB. **F,** Western blot of several mitophagy regulators in GPR176 knockdown cells. **G,** mRNA expression of BNIP3L and PINK1 in GPR176 knockdown cells. The results of HCT116 were shown here. **H,** Knockdown efficiency of BNIP3L in CRC cells assessed by qPCR and western blot. **I,** Western blot of mitophagy proteins and BNIP3L phosphorylation levels in GPR176 knockdown cells treated with BNIP3L plasmid. **J,** Confocal immunofluorescence analysis in GPR176 knockdown cells treated with BNIP3L plasmid. Data was presented with Mean±SD,**$p < 0.01$.

Supplementary Figure 5. GPR176/GNAS complex regulates cell proliferation *in vitro* and *in vivo*.

The knockdown and overexpression efficiency of GANS in CRC cells assessed by **A,** qPCR and **B,** western blot. **C,** Co-IP assay in 293T cells and CRC cells. **D,** CCK-8 assay and **E,** plate colony in GPR176 overexpressed cells transfected with shGNAS. **F,** Gross images of xenograft model in GPR176 overexpressed cells transfected with shGNAS *in vivo*. **G-H,** Statistical results of xenograft model in GPR176

overexpressed cells transfected with shGNAS plasmid respectively. **I-J,** Plate colony and CCK-8 in GPR176 knockdown cell transfected with GNAS or vector control. Data was presented with Mean±SD, ***$p < 0.001$, **$p < 0.01$ and *$p < 0.05$, ns indicated no significance.

Supplementary Figure 6. Effect of GPR176/GNAS complex on CRC progression depends on their interaction.

A, Western blot of GNAS level in GPR176 knockdown cells treated with GPR176 WT or GPR176 Mut2 plasmid. **B,** mRNA level of GNAS in GPR176 knockdown cells. **C,** Western blot of mitophagy proteins in GNAS overexpressed cells treated with GPR176 WT or GPR176 Mut2 plasmid. **D-E,** Flow cytometry analysis of cell cycle and apoptosis in endogenous GPR176 knockdown cells and exogenous GPR176 OE cells treated with GNAS plasmid. **F-G,** CCK-8 assay and plate colony assay in endogenous GPR176 knockdown cells and exogenous GPR176 OE cells treated with GNAS plasmid.

Supplementary Figure 7. Specificity of GPR176 in CRC.

A, Immunofluorescence analysis of LC3B in GPR176 knockdown and overexpressed normal cells. **B,** Expression of GPR176 in bladder cancer, breast cancer and lung cancer.
